# Graph-theoretical analysis for energy landscape reveals the organization of state transitions in the resting-state human cerebral cortex

**DOI:** 10.1371/journal.pone.0222161

**Published:** 2019-09-09

**Authors:** Jiyoung Kang, Chongwon Pae, Hae-Jeong Park

**Affiliations:** 1 Center for Systems and Translational Brain Sciences, Institute of Human Complexity and Systems Science, Yonsei University, Seoul, Republic of Korea; 2 Department of Nuclear Medicine, Yonsei University College of Medicine, Seoul, Republic of Korea; 3 BK21 PLUS Project for Medical Science, Yonsei University College of Medicine, Seoul, Republic of Korea; 4 Department of Cognitive Science, Yonsei University, Seoul, Republic of Korea; University of Texas at Austin, UNITED STATES

## Abstract

The resting-state brain is often considered a nonlinear dynamic system transitioning among multiple coexisting stable states. Despite the increasing number of studies on the multistability of the brain system, the processes of state transitions have rarely been systematically explored. Thus, we investigated the state transition processes of the human cerebral cortex system at rest by introducing a graph-theoretical analysis of the state transition network. The energy landscape analysis of brain state occurrences, estimated using the pairwise maximum entropy model for resting-state fMRI data, identified multiple local minima, some of which mediate multi-step transitions toward the global minimum. The state transition among local minima is clustered into two groups according to state transition rates and most inter-group state transitions were mediated by a hub transition state. The distance to the hub transition state determined the path length of the inter-group transition. The cortical system appeared to have redundancy in inter-group transitions when the hub transition state was removed. Such a hub-like organization of transition processes disappeared when the connectivity of the cortical system was altered from the resting-state configuration. In the state transition, the default mode network acts as a transition hub, while coactivation of the prefrontal cortex and default mode network is captured as the global minimum. In summary, the resting-state cerebral cortex has a well-organized architecture of state transitions among stable states, when evaluated by a graph-theoretical analysis of the nonlinear state transition network of the brain.

## 1 Introduction

A dynamic complex system can possess several stable states (attractors) for a given set of system parameters [1–6]. If a system has multiple coexisting stable states and can switch among them in response to noise or intrinsic perturbations to the system, it is generally referred to as a multistable system [7, 8]. In this respect, the brain at rest can be considered as a system showing multistability [1–7, 9, 10]. From the perspective of the multistable brain, the conventional term “resting state” is not a homogeneous state but a period of switching among multiple micro-states (or sub-states). Here, we will refer to a brain state as a sub-state during the resting-state period. Of note, this multistablity perspective differs from studies on functional connectivity dynamics [11–20], which have described the dynamic nature of the brain in terms of temporal changes in its interactions (connectivity parameters). In contrast, multistability in the complex system is an emergent property of nonlinear interactions among nodes in the system without any changes in their connectivity.

**Fig 1:**
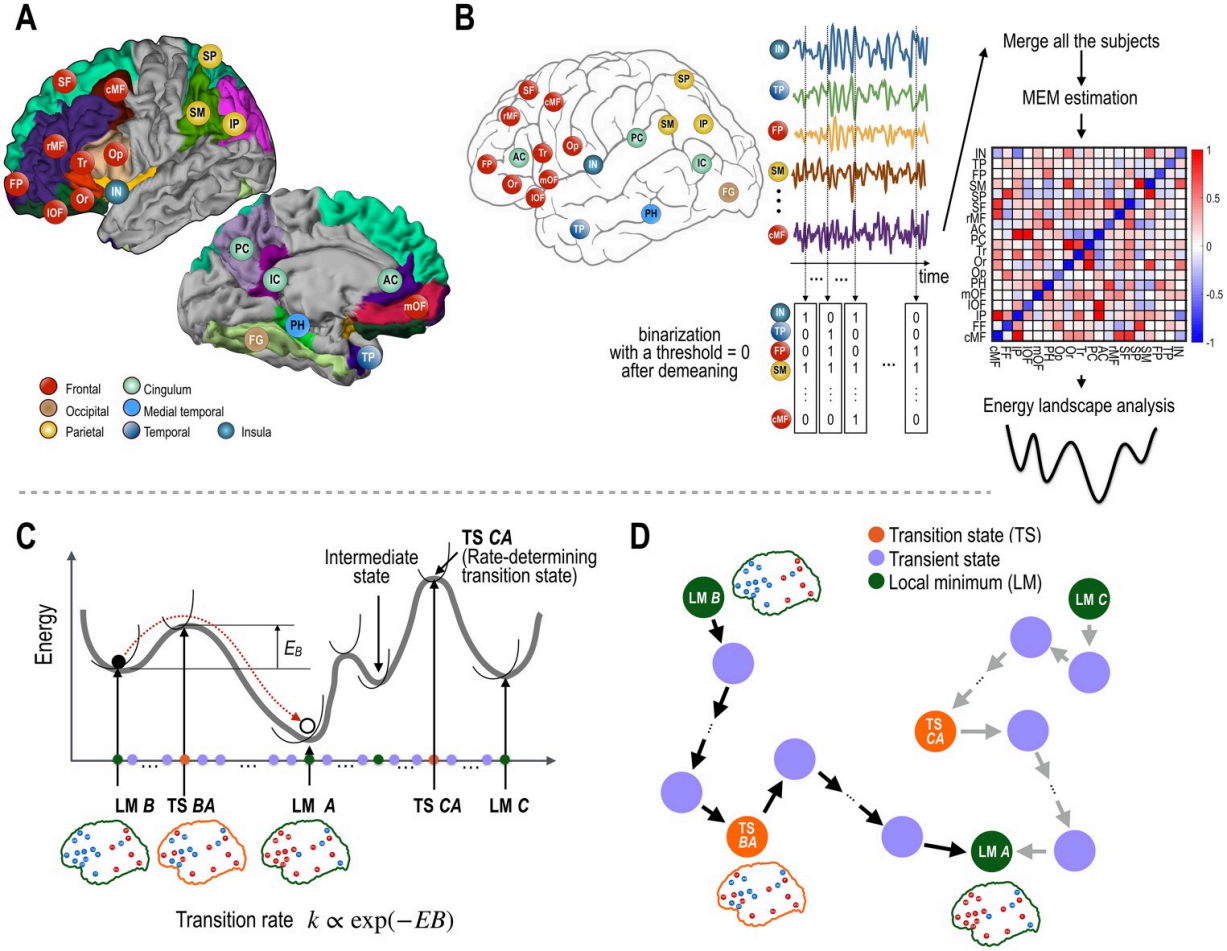
Procedures of the present study. (A) Regions of interest (ROIs) in the human cerebral cortex are shown. (B) Functional magnetic resonance imaging (fMRI) data of the resting-state were binarized to represent brain states (active or deactive). These binarized states were used to construct the pairwise maximum entropy model, which was used to construct the energy landscape. (C, D) An illustration of the construction of the state transition network is presented. Local minima (LM) and transition states (saddle points, TS) on the transition pathway in the energy landscape (C), were used as nodes in the transition network as shown in (D). For each pathway, a transition rate was assigned as the weight on its edges (steps on the pathway) of the state transition network. Among transition states along the path, the transition state with the highest energy on the pathway determines the transition rate (called rate-determining transition state). Therefore, for simplicity, we refer to the rate-determining transition state as the TS for the transition between two states. LM A, LM B, LM C indicate local minima and TS BA indicates the transition from B to A, and TS CA, the transition from C to A. E_B_ indicates the energy barrier in the transition path. The green, purple, and orange color nodes represent local minimum, transient, and transition state nodes, respectively. The colors of the nodes in the brain maps indicate active (red) and deactive (blue) regions.

In a multistable system, stable states and the transition processes among them characterize the dynamics of the system. To explore the multistability and state transitions in the dynamic brain, energy landscape analysis has recently been applied to fluctuations of blood oxygenation level-dependent (BOLD) functional magnetic resonance imaging (fMRI) [21–27]. Prior to its introduction to the brain research field, energy landscape analysis had already shown its utility in understanding the dynamics of multi-dimensional complex systems, such as protein dynamics and the thermodynamics of liquids [28–33]. In the studies of brain dynamics using energy landscape analysis, distributed activity patterns across brain regions have often been used to define brain states, one of which the brain belongs to at each measurement time point [21–27]. In the energy landscape analysis, the energy of a state is the negative log probability of the occurrence of the state (thus, frequent states have low energy) according to the Boltzmann distribution of the state. The (inverse) frequency distribution of all possible brain states (patterns of brain activities across the brain regions) is called an energy landscape (see Fig 1).

The energy landscape of the system consists of several valleys with local minima (called “stable states” or “attractors”, abbreviated as LM) that have energies lower (more frequent) than their neighbors do in the valleys. Thus, the dynamics of the system can be divided into intravalley (within the basin of a local minimum) and intervalley (between local minima) motions. In the former case, a state of the system wanders around a local minimum of the energy surface that the state belongs to, whereas in the latter case, a state transits from one local minimum to another, surpassing an energy barrier.

For a state transition between two local minima states, an optimal pathway refers to the path with the lowest maximal energy barrier among all possible paths. The optimal path may contain “intermediate states” (a type of local minima) and “transition states” (saddle point states) along the path. Among many transition states along the path, the transition state with the highest energy on the pathway determines the transition rate. Therefore, for brevity, we refer to this rate-determining transition state (having the highest energy on the pathway) as the transition state (TS) between two states (See Fig 1). Table 1 summarizes the terminology used in the current paper.

**Table 1:** Definitions of terminologies.

Terminology	Definition in this study
State, *V*_*i*_	A distributed brain activity pattern (a 19-dimensional binarized vector)
Stable state	A local minimum in the energy landscape, a brain activity pattern that sustains itself, and returns to its state following little perturbations. A state *V*_*i*_ is a stable state, if it satisfies, *E*(*V*_*i*_) < *E*(*V*_*nei*_), where *V*_*nei*_ are all neighbor states and *E*(*V*_*i*_) is the energy of the state *V*_*i*_.
Neighbor state	A states *V*_*j*_ is a neighbor state for a state *V*_*i*_, if *d*(*V*_*i*_, *V*_*j*_) = 1, where *d*(*V*_*i*_, *V*_*j*_) represents Hamming distance, i.e., the number of different elements (regional activity) in the vectors of two brain states *V*_*i*_ and *V*_*j*_.
Hub state	A highly connected (high node degree) state in the state transition network, indicating frequently occurring (or visiting) states while transitioning among different brain states.
Rate-determining transition state (TS)	When a transition path contains more than one local minimum on the path (except for initial and final local minima), the transition path has multiple transition states (saddle points). The highest energy indicates the least occurrence. Among multiple transition states, the state with the highest energy determines the transition rate of transitioning between two states. The rate-determining transition state is referred to as the transition state (TS) of the transition path between two states in the current study.
Intermediate state	A stable state on a transition path from a state to the other state, except for the initial and final states of the transition path.
Energy	Minus log (occurrence) probability of a state. Probability of appearance at a low energy state is higher than those of high energy states. In this study, the probability distribution of the states follows the Boltzmann distribution, which is given by Eq 5.
Transition path	The path that has the minimal energy barrier among all possible paths from a local minimum to the other local minimum.
Transition rate	Transition speed from a local minimum to the other local minimum, defined as *exp*(-*E*_*B*_) based on the state transition theory. *E*_*B*_ is the energy barrier which is the energy difference between the highest energy on the transition path and the energy of the initial state.
STN	State transition network (STN) composed of brain states as nodes and transition rates as edges.
STN-FS	A full state transition network composed of all possible states along transition pathways between all pairs of stable states (local minima).
STN-GM	A state transition network composed of all states on the path from all local minima toward the global minimum.
STN-LM	A state transition network composed of rate-determining transition states and local minima.

Despite a growing number of studies on the multistability of the resting brain systems [1, 3–6, 21, 34–37], the state transition processes between local minima in the brain systems have not yet been sufficiently investigated. In the present study, we explored the multistability and state transitional properties of the human cerebral cortex system. We estimated an energy landscape of brain states using a pairwise maximum entropy model (MEM) of the resting-state fMRI (rs-fMRI) data from the Human Connectome Project (HCP) database [38]. We extracted local minima and optimal pathways among them in the energy landscape, and then explored the characteristics of the brain state transition processes from the network-theoretical perspective using the state transition network, where states are represented as nodes, transitions between two states (nodes) as edges, and transition rates as edge weights.

We analyzed the state transition network using the degree of state nodes (occurrence frequency during state transition) and path lengths (how many transient states are needed to arrive at a final state). From this transition network analysis, we also tested whether hub-like TSs exist, similar to spatial hubs found in the conventional network analysis of the brain [39–42]. We further examined state transitions toward the global minimum to identify intermediate stable states (a type of local minima) that mediate those multi-step transitions. The hierarchy in the brain state transition was investigated by clustering state transitions into intra- and inter-group transitions according to the transition rate. We finally investigated the organizational properties of the resting-state brain by comparing the transitional properties of the baseline cortical system with those of a virtual system by altering the MEM parameters.

The results of our analysis suggest that the cerebral cortex system at rest contains multiple stable states that are clustered into two major state groups. The transition between brain states across the two state groups was mediated by a frequent TS, which operated as a hub of the transition network. When we removed this hub state, which bridges most transition processes across the two groups, between-group transitions occurred via an alternative TS, indicating redundancy in state transition. State transition in the brain appears to involve multi-step state transitions, with some stable states serving as intermediate states for the complete transition. We also found that the baseline cerebral cortex at rest shows a more complex and organized state transition network than those of artificially altered systems. This network approach to the state transition in the brain may provide a new framework for brain exploration and become an effective tool for understanding healthy and abnormal brain systems, concerning brain state dynamics.

## 2. Materials and methods

### 2.1 Resting-state fMRI data set

The pairwise MEM (explained in the following section) was generated using rs-fMRI data of 470 participants (192 males, 278 females, age: 29.19 ± 3.51 years) from the HCP database [38], which was used in our previous study [21]. Briefly, all data were sampled at TR = 0.72 s, during 4 runs, with 1200 time points per run. We projected the voxel-wise time courses from within a region on the eigenvector linked to the largest eigenvalue (obtained using principal component analysis) to generate the time series of interest for each region.

The effects of rigid motion and their derivatives were regressed out, followed by linear detrending and despiking of the extracted signals [43–46]. Although there is an ongoing debate concerning filtering and global regression, we regressed out global signal changes in the whole-brain mask to emphasize local and short-term state fluctuation (high or low) at each region in representing specific brain states, followed by a high-pass filtering at 0.01 Hz. Indeed, the current analysis is based on the assumption of the resting-state being in an equilibrium, i.e., without long-term statistical (temporal) changes in the dynamic properties.

Since computational cost dramatically increases with the number of degrees of freedom of the system (2^N^, N: number of nodes), we extracted the rs-fMRI time series of only 19 regions of interest (ROIs) out of 33 cortical regions defined in the Desikan-Killiani Atlas [47]. In choosing 19 ROIs to define a cortical state, we included brain regions associated with the default mode network (DMN), the salience network, the parietal lobe and prefrontal cortices to focus on resting-state dynamics at the higher cognitive brain areas [48] in the cognitive hierarchy. Due to the complexity, we excluded primary/secondary sensory, motor cortical regions and subcortical brain regions in the evaluation. We also confined ROIs to a hemisphere since previous studies showed strong symmetric activities (e.g., symmetric independent components found in many previous studies, including Smith, Beckmann [49]) and strong interhemispheric connectivity; e.g., Kang, Pae [21]. Coactive regions across hemispheres exhibit similar time courses and, thus, are considered to be less informative in defining diverse brain states.

The ROIs used in this study are the precuneus (PC), parahippocampal gyrus (PH), caudal middle frontal gyrus (cMF), fusiform gyrus (FG), inferior parietal lobe (IP), isthmus cingulate gyrus (IC), lateral orbitofrontal gyrus (lOF), medial orbitofrontal (mOF), pars-opercularis (Op), pars-orbitalis (Or), pars-triangularis (Tr), rostral anterior cingulate gyrus (AC), rostral middle frontal gyrus (rMF), superior frontal gyrus (SF), superior parietal gyrus (SP), supramarginal gyrus (SM), frontal pole (FP), temporal pole (TP), and insula (IN) in the left hemisphere (Fig 1A). The ROIs in the left-hemisphere were mainly evaluated and presented in the current study because the default mode network and many frontal cortices of interest have stronger connections (hubs) in the left hemisphere than the right hemisphere [50]. However, we confirmed that similar results were obtained from the ROIs in the right-hemisphere (see Section B in S1 File).

For each ROI, signals were thresholded to represent deactive (0) and active (1) states. Since the number of local minima was maximized when the threshold was zero in the empirical evaluation (unpresented) and in our previous analysis [21], we selected zero as the threshold to binarize regional states after global regression (Fig 1B). A brain state was defined by merging all (binarized) 19 regional states into a state vector (the number of elements of a state vector is 19). Due to a high sample size demand to estimate brain states (for all 2^19^ possible states), we concatenated all brain state samples from four sessions of 470 participants into a group-level sample data set (the total number of state samples, 1200 samples × 4 sessions × 470 participants) and estimated parameters of the group-level pairwise MEM using the method described in the following section.

### 2.2 Construction of pairwise maximum entropy model (MEM)

To analyze resting-state activity in the cerebral cortex, we utilized the pairwise MEM estimation approach described in previous studies (Fig 1B) [21–23].

The estimation process consists of a step for defining brain states and a pairwise MEM model for state dynamics, and an optimization step for MEM model parameters to fit probability distributions of empirical brain states and states generated by the model. Details of the model construction are provided in our previous study [21] (for the mathematical details, see review ref. [51]).

Suppose the brain state space is represented by

$$S=\{V=[\sigma_{1},\ldots ,\sigma_{N}{]}^{⊤}\in \{0,1{\}}^{N}\}V\;\text{is}\;\text{a}\;\text{possible}\;\text{state}\},$$ (1)

where the value of *σ*_*i*_(*i* = 1, 2, …, *N*) is either 0 (deactive) or 1 (active), indicating a local activity at a node (brain region) *i*. *N* denotes the total number of nodes (or ROIs). For a probability distribution *p*(*V*_*k*_) for all possible brain states *V*_*k*_(*k* = 1, 2, …, 2^N^), the entropy *S* can be defined as

$$S=-\sum_{k=1}^{2^{N}}p(V_{k})\text{ln}\;p(V_{k}).$$ (2)

In the pairwise MEM, the average of each node activity,

$$\langle \sigma_{i}\rangle =\frac{1}{T}\sum_{t=1}^{T}\sigma_{i}(t),$$ (3)

and the averages of all pairwise products,

$$\langle \sigma_{i}\sigma_{j}\rangle =\frac{1}{T}\sum_{t=1}^{T}\sigma_{i}(t)\sigma_{j}(t),$$ (4)

derived from the experimental data (i.e., *σ*_*i*_(*t*), *σ*_*j*_(*t*)), play as constraints that should be fit in the model estimation. With these constraints, maximizing the entropy *S* derives the probability *p*(*V*_*k*_) of a brain state *V*_k_ as a Boltzmann distribution

$$p(V_{k})=\frac{e^{-E(V_{k})}}{\sum_{l=1}^{2^{N}}e^{-E(V_{l})}},$$ (5)

where *E*(*V*_k_) indicates the energy of the state *V*_k_ and can be described as the following equation:

$$E(V_{k})=-\sum_{i=1}^{N}H_{i}\;\sigma_{i}(V_{k})-\sum_{i=1}^{N}\sum_{j>i}^{N}J_{ij}\sigma_{i}(V_{k})\sigma_{j}(V_{k}),$$ (6)

where the parameters *H*_i_ and *J*_ij_ represent the activation tendency (baseline sensitivity) of node *i* and the pairwise interaction between nodes *i* and *j*, respectively. *σ*_*i*_(*V*_*k*_) indicates an activity at a node *i* (i.e., *σ*_*i*_) in a brain state *V*_*k*_. A gradient ascent algorithm was employed to estimate MEM parameters, *H*_*i*_ and *J*_*ij*_. These parameters were iteratively updated to minimize the difference of average values in Eqs (3) and (4) between model-generated signals (with parameters) and observed signals using the following equations,

$$H_{i}^{new}=H_{i}^{old}+\alpha_{g}log\frac{\langle \sigma_{i}\rangle }{{\langle \sigma_{i}\rangle }_{m}},$$ (7)

$$J_{ij}^{new}=J_{ij}^{old}+\alpha_{g}log\frac{\langle \sigma_{i}\sigma_{j}\rangle }{{\langle \sigma_{i}\sigma_{j}\rangle }_{m}}.$$ (8)

Here, 〈*σ*_*i*_〉_*m*_ and 〈*σ*_*i*_*σ*_*j*_〉_*m*_ were calculated as follows:

$${\langle \sigma_{i}\rangle }_{m}=\sum_{k=1}^{2^{N}}\sigma_{i}(V_{k})p(V_{k}),$$ (9)

$${\langle \sigma_{i}\sigma_{j}\rangle }_{m}=\sum_{k=1}^{2^{N}}\sigma_{i}(V_{k})\sigma_{j}(V_{k})p(V_{k}).$$ (10)

The scale parameter *a*_*g*_ was initially set to 0.1. The parameters were optimized until the gradients reached a value lower than 10^−5^.

To evaluate the effectiveness of the pairwise MEM, we calculated the accuracy value, *r*_*D*_,

$$r_{D}=\frac{\left(D_{1}-D_{2}\right)}{D_{1}}.$$ (11)

Here, *D*_*k*_ is the Kullback-Leibler divergence between the probability distributions of the *k*-th order model network and the empirical network,

$$D_{k}=\sum_{l=1}^{2^{N}}p_{N}(V_{l})log_{2}\frac{p_{N}(V_{l})}{p_{k}(V_{k})},$$ (12)

where *p*_*N*_ represents the empirical distribution of the network state. In the calculation for the empirical probability distribution of brain states, we calculated the frequency of each state in the group-level sample data set described above.

We also evaluated the reliability parameter *ER*,

$$ER=\frac{r_{S}}{r_{D}}.$$ (13)

Here, *r*_*s*_ and *S*_*k*_ are given by

$$r_{S}=\frac{\left(S_{1}-S_{2}\right)}{\left(S_{1}-S_{N}\right)},$$ (14)

$$S_{k}=-\sum_{l=1}^{2^{N}}p_{k}(V_{l})log(p_{k}(V_{l})).$$ (15)

The measures *r*_*D*_ and *r*_*S*_ evaluate the adequacy of the pairwise MEM over the independent MEM in explaining time series, in two different aspects; *r*_*D*_ and *r*_*S*_ use Kullback-Leibler divergences and difference of the entropy between independent (1st order) and pairwise (2nd order) MEMs. The reliability *ER* was defined to compare those two different measures. If *H*_*i*_ and *J*_*ij*_ are estimated without error, *ER* is equal to 1.

### 2.3 Energy landscape analysis

To describe the dynamics of the cerebral cortex system at rest, we performed the energy landscape analysis. More specifically, first, we elucidated the local minima (attractors), and then evaluated energy barriers between pairs of attractors, following the procedure described in previous works [21, 23].

To construct an energy landscape, the distance between two states should be first defined. Based on this distance, neighbor states can be defined to extract local minima. Following previous energy landscape studies, we defined the distance between two states as the number of elements (bits) that differ between two state vectors. We also assumed a gradual state transition, and the energy landscape was examined by changing one element of the state vector for each step.

The local minima (also called stable states) were defined as states that have lower energy (more frequent) relative to their neighbors. To evaluate the energy barrier for each local minima pair, the lowest energy pathways were extracted by using disconnectivity graph analysis [52]. Specifically, for each possible pair of local minima, we recorded the shortest path connecting the two local minima. The highest energy on this path was selected as a threshold to remove states that exhibited higher energy than the threshold. We repeated this step until the two local minima had been disconnected. The highest energy value of the last connected path was assigned to the threshold of the local minimum pair. The energy barrier, *E*_*B*_, between two local minima *i* and *j*, was defined as the lower value between *E*_*th*_(*V*_*i*_,*V*_*j*_)–*E*(*V*_*i*_) and *E*_*th*_ (*V*_*i*_,*V*_*j*_) − *E*(*V*_j_), where *E*_*th*_ (*V*_*i*_,*V*_*j*_) represents the threshold as defined above. These disconnectivity graph calculations were performed using the i-graph library [53].

### 2.4 Construction of the state transition networks

In the present study, we constructed three types of state transition networks; a full state transition network composed of all possible states along transition pathways among local minima (STN-FS), a state transition network of states from local minima toward the global minimum (STN-GM), and a state transition network among TSs and local minima states (STN-LM).

**Fig 2:**
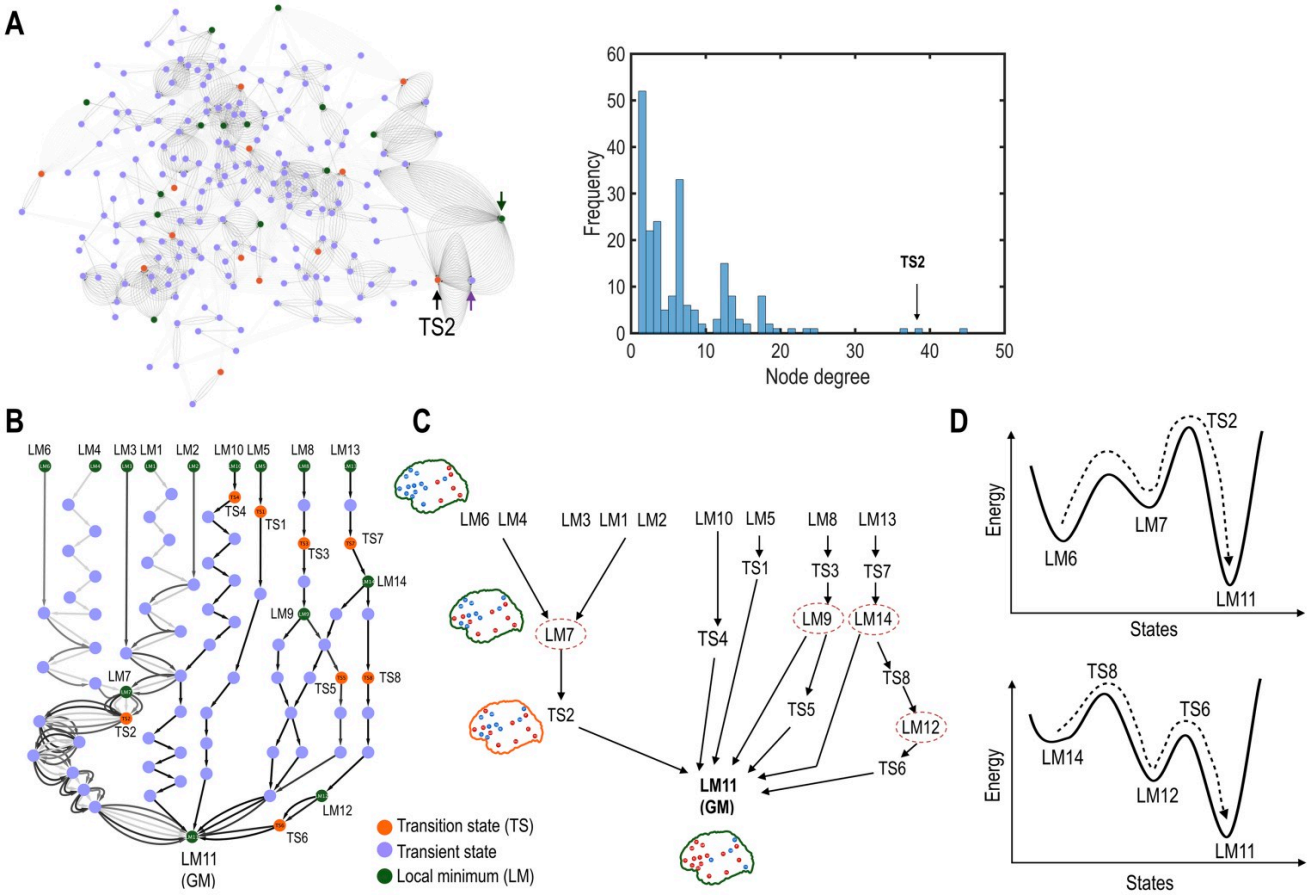
Analysis of the state transition networks. (A) The state transition network among full states (STN-FS) is shown in the left panel. We assigned all states in the state transition process to the nodes. The nodes with high node degrees are designated with arrows. The right panel shows a histogram of node degrees for all states (nodes) in the STN-FS. A transition state, TS2, has a significantly high node degree. (B, C) State transition processes (STN-GM) from local minima (LM) toward the global local minimum (LM11) is shown in (B). An illustration of the state transition processes in STN-GM is presented in (C). (D) Two representative examples, state transitions from LM6 to LM11 (upper panel) and from LM14 to LM11 (bottom panel), are shown. TS represents a transition state (a saddle point). The green, purple, and orange colors represent local minimum, transient, and transition states, respectively. Intermediate states are encircled.

We first constructed a STN-FS as a directional weighted network (Fig 2A). For all possible pairs of local minima, state transition pathways were identified as described in the above section. All states on the state transition pathways among local minima were considered nodes of the STN-FS. Since the forward and backward state transition pathways were identical for a pair of local minima, we only considered the state transitions from the higher to lower local minimum. For all edges (transitions between pairs of states), we assigned weights using a transition rate, which is given by

$$exp\left(-E_{B}\right).$$ (16)

Here, *E*_B_ represents the energy barrier (or activation energy) which is the energy difference between the highest energy on the transition path and the energy of the initial state (see Fig 1C).

We then constructed the STN-GM to focus on the details of the state transition processes toward the global minimum (Fig 2B). To construct this network, all nodes and edges that were not connected to the global minimum in the STN-FS were removed. In the STN-GM analysis, we particularly identified intermediate states that mediate multi-step transitions toward the global minimum.

**Fig 3:**
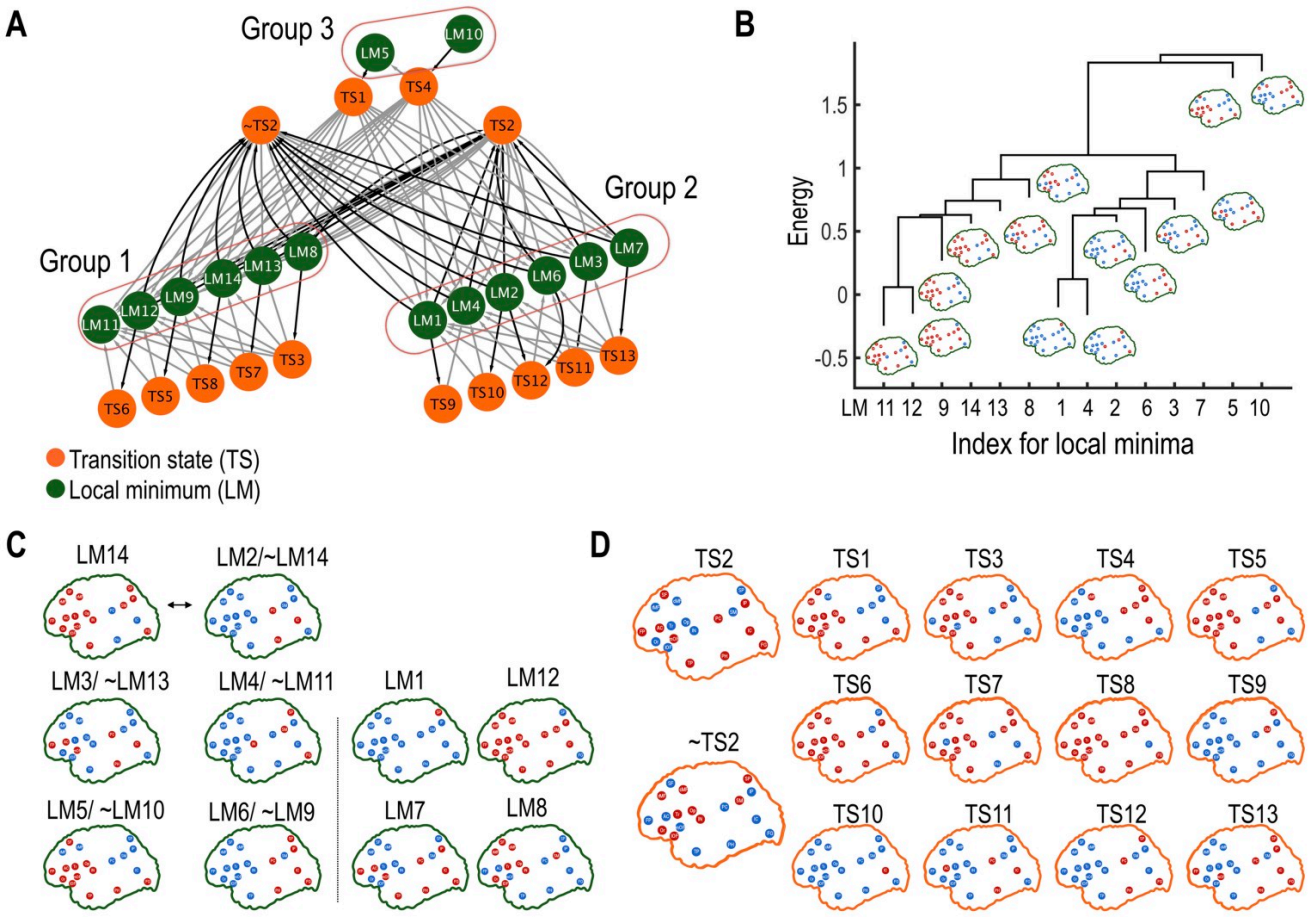
Analysis of the state transition network (STN-LM) composed of rate-determining transition states (TS) and local minima states (LM). (A) The STN-LM is shown. Black and gray colored lines represent an inward direction to and an outward direction from the TS. (B) Local minima (LM) were clustered according to energy barriers. The leaf ends of the dendrogram represent the energy values of the corresponding local minima. (C) Activation patterns of the local minima. The “~” sign represents complementary states. For instance, LM2/~LM14 indicates that LM2 and LM14 are each other’s complementary states. (D) Activity patterns of the transition states are shown with TS2 and ~TS2 as major hub transition states. The red and blue dots represent the active and deactive states of the ROIs. The green and orange colors represent local minima and transition states.

In order to focus on the transition rate among local minima, we constructed a STN-LM (Fig 3A), considering local minima and TSs as nodes and transition rates as edges. According to the transition state theory, a transition rate between two states is determined solely by the energy difference of the initial state and the rate-determining transition state (saddle point), i.e., the TS (Fig 1C). In other words, the transition rate between two states only depended on its energy barrier (maximal energy difference) along the pathway.

Based on the transition rates in STN-LM, we evaluated the architecture of the state transitions of the system by applying a cluster analysis of transition rates to differentiate intra- and inter-group transitions. The clustering of transitions in the STN-LM was conducted using the UPGMA (Unweighted Pair Group Method with Arithmetic Mean) algorithm [54] with Euclidian metric; distances between nodes were defined by transition rates.

The constructed network was analyzed in terms of network theory using node degree (frequency of occurrence during state transition) and path length (number of transitions to reach the target state). We also analyzed the effective path length between local minima, which is defined as the difference between the total path length (number of transitions) and the Hamming distance (number of different elements between two state vectors, i.e., number of regions that showed active/deactive differences) of the initial and final states.

We finally investigated the organizational properties of the resting-state brain by comparing the transitional properties of the baseline cortical system with those of a virtual system by altering the MEM parameters. The comparison between the resting-state brain and the altered (virtual) system was conducted over the STN-FS and STN-LM.

## 3 Results

### 3.1 Maximum entropy model for the cerebral cortex system at rest

In order to generate the energy landscape of the brain state, we estimated the first and second order interaction parameters (i.e., baseline sensitivity *H*_*i*_ and pairwise interactions *J*_*ij*_) of the MEM using binarized rs-fMRI activation patterns. The activation patterns of rs-fMRI data were reproduced with a high accuracy of fit (*r*_*D*_ = 86.3%) and reliability (*ER* = 99.9%) (Figure A in S1 File). Baseline sensitivity parameters *H*_*i*_ and pairwise interaction, *J*_*ij*_, are displayed in Figure A in S1 File and Fig 1B. Details of the obtained MEM parameters are described in Figure A in S1 File.

### 3.2 Multiple stable states in the resting-state of the cerebral cortex system

Analysis of the energy landscape identified 14 local minima of the cerebral cortex system at rest (Figs 2 and 3). Complementary states (active versus deactive for each brain region) of five local minima were also found to be local minima (Fig 3). Two pairs, local minimum (LM) 1 and LM12, LM7 and LM8, were nearly complementary states of each other. In these pairs, all regions were complementary except for one brain region, in each: the deactive precuneus (PC) in the LM7 and LM8, and deactive fusiform gyrus (FG) in the LM1 and LM12.

The most stable local minimum (i.e., global minimum) was LM11, where most cortical regions were active except for the insula, supramarginal gyrus, superior parietal lobe, and fusiform gyrus (see LM11 map in Fig 2C).

### 3.3 The state transition network among full states (STN-FS)

Utilizing disconnectivity graph analysis [52], 91 transition pathways were extracted for all possible pairs of the 14 local minima. All states on the 91 transition pathways were regarded as nodes and transition rates between pairs of nodes as edges in the STN-FS (Fig 1C and 1D). As a result, a total of 219 nodes and 1201 edges composed an STN-FS (Fig 2A). When we evaluated node degrees for all nodes in the STN-FS, three (state) nodes showed a significantly higher node degree than the rest (arrows in Fig 2A). Most (86.8%) effective path lengths, i.e., the difference between the total path length and the Hamming distance of two initial and final local minima, were less than 8 (Figure B in S1 File). For half of the total number of pathways (49 pathways), effective path lengths had the shortest value of 0.

### 3.4 Analysis of state transition network from local minima toward the global minimum (STN-GM)

For 14 local minima, 13 transition processes toward the global minimum were considered in the STN-GM with 82 nodes and 141 edges. A total of eight TSs, which determine transition rate, were found in the STN-GM. The STN-GM also showed that intermediate local minima (e.g., LM7, LM9, LM12, and LM14) were involved in the transition processes of other local minima transitioning toward the global minimum (Fig 2B and 2C). For instance, the transition pathways that started from the LM1, LM3, LM4, LM6, and LM12 passed through LM7 before reaching the global minimum (LM11). The rates for these transitions were determined by energy differences between TS2 and the initial local minima. The state transition from LM6 to the global minimum (LM11) contained an intermediate state (LM7) and the energy of the rate-determining transition state between LM6 and LM7 was smaller than that of LM7 and LM11 (i.e., energy of TS2), and, thus, the rate-determining transition state was TS2 (upper Fig 2D).

However, LM5, LM8, LM9, LM10, LM12, LM13, and LM14 had their own rate-determining transition states along transition paths toward the global minimum (Fig 2B and 2C). Indeed, in the state transition from LM14 to the global minimum (LM11), which contained an intermediate state (LM12), the energy of the rate-determining transition state between LM11 and LM12 (energy of TS8) was larger than that of LM12 and LM11 (energy of TS6), and, thus, the rate-determining transition state was TS8 (lower Fig 2D).

In this way, by analyzing this reduced state transition network (STN-GM), we could identify the characteristics of all transition processes on their way to the global minimum.

### 3.5 Analysis of a state transition network among rate-determining transition states and local minima states (STN-LM)

The STN-LM was composed of 27 nodes (13 TSs plus 14 local minima) and 90 edges (Fig 3A). We found a clustered structure in the STN-LM: one cluster containing six local minima (LM8, LM9, LM11, LM12, LM13, and LM14), and the other containing their complementary local minima (LM1, LM2, LM3, LM4, LM6, and LM7). A similar clustering result was found by using energy barriers (Fig 3B). Interestingly, only one rate-determining transition state, TS2, bridged two clusters. TS2 is composed of active regions in the FP, SF, AC, mOF, PC, IP, IC, TP, PH, FG, which overlap mostly with coactivation of the default mode network [48] and the anterior and medial temporal lobe (Fig 3D). Since TS2 has a high node degree (Fig 2A), we can refer to TS2 as a hub in the transition network.

To investigate the effects of TS2 on the transition process, we removed TS2 and explored the state transition pathways. After the removal of TS2, we found 36 alternative pathways. In these 36 pathways, the complementary state of TS2, namely ~TS2, bridged the state transition processes between clusters, instead of TS2. The energy difference between TS2 and ~TS2 was very small, 0.00737. Since the transition rate is proportional to *exp*(-*E*_barrier_) in state transition theory, the estimated ratio of the transition rate between the original and alternative pathway was 99.27%. Thus, we added the ~TS2 node to the state transition network, STN-LM. Moreover, the property of clustered transitions was also observed for the transition processes in the ~TS2 system (Figure C in S1 File). Since TS2 and ~TS2 have high node degrees (i.e., measure of frequency of appearance), both TS2 and ~TS2 play as hub TSs.

**Fig 4:**
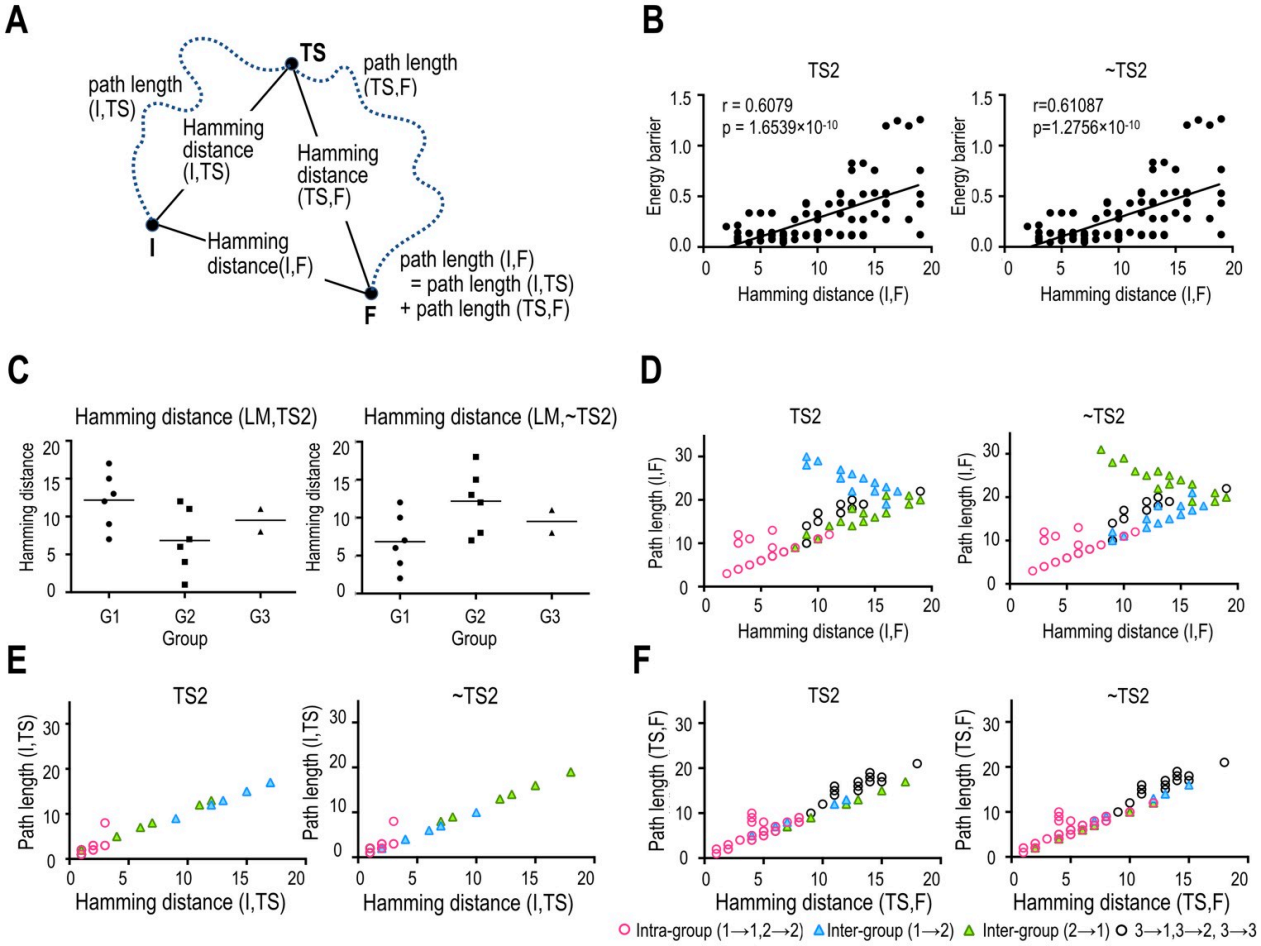
Path lengths and Hamming distances of the state transitions in the TS2 and ~TS2 systems. The ~TS2 system indicates transition pathways when the TS2 node is removed. (A) Definitions of the path lengths from the initial (I) to the final state (F) with transition state (TS) are presented. (B) Positive correlations between the energy barriers and Hamming distances of the initial and final states are presented. (C) Hamming distances between the local minima and TS2 (or ~TS2) are presented for each group. (D) Full path lengths are plotted according to Hamming distances of the initial and final states. (E, F) Path lengths between initial and transition states (E), and path lengths between final and transition states (F) are plotted according to their Hamming distances. The definitions of the groups (G1, G2, and G3) are presented in Fig 3A. In (B) and (D)—(F), the left and right panels represent the results of the TS2 and ~TS2 systems. The red points represent intra-group state transitions (i.e., transitions of group 1 → 1 and group 2 → 2). The blue and green triangle points represent the results of the state transitions between the groups; group 1→2 (blue) and group 2→1 (green).

We extracted the factors that determined transition rates (i.e., energy barriers) for both the TS2 system and TS2 removed (~TS2) system (Fig 4); the Hamming distances between initial and final states were positively correlated with energy barriers (r = 0.608, p = 1.654 × 10^−10^ for the TS2 system, and r = 0.611 p = 1.276 ×10^−10^ for the ~TS2 system, Fig 4B). However, there was no such relation between the energy barriers and effective path lengths (Figure C in S1 File).

We further investigated the transition processes by separating the inter-group and intra-group processes (Fig 4D, 4E and 4F). For the intra-group transitions, positive correlations between Hamming distances and path lengths were observed for both TS2 and ~TS2 systems.

However, for the inter-group transitions, we could not find such associations. Thus, we further separated the inter-group transitions and found negative and positive correlations between Hamming distances and path lengths for the transitions from group 1 to 2 (r = -0.865, p = 3.123 × 10^−5^), and from group 2 to 1 (r = 0.921, p = 3.240 × 10^−9^) in the TS2 system (Fig 4D).

Interestingly, in the ~TS2 system, the correlations were reversed; positive and negative correlations were found for the transitions from group 1 to 2 (r = 0.865, p = 3.123 × 10^−5^), and from group 2 to 1 (r = -0.921, p = 3.240 × 10^−9^) (Fig 4D). Since the distances between TS2 and local minima of group 1 were longer than those of group 2 in the TS2 system, but in the ~TS2 system, an inverse association was found (i.e., the distances between ~TS2 and local minima of group 1 were shorter than those of group 2) (Fig 4C), the cause of the inverse correlations could be the distance between the transition state and initial states. In the inter-group transitions, path lengths from an initial local minimum to the other group local minima depended on Hamming distances from the initial local minimum to the hub TS (Fig 4E and 4F).

### 3.6 Effects on the state transition processes following the alteration of the system

**Fig 5:**
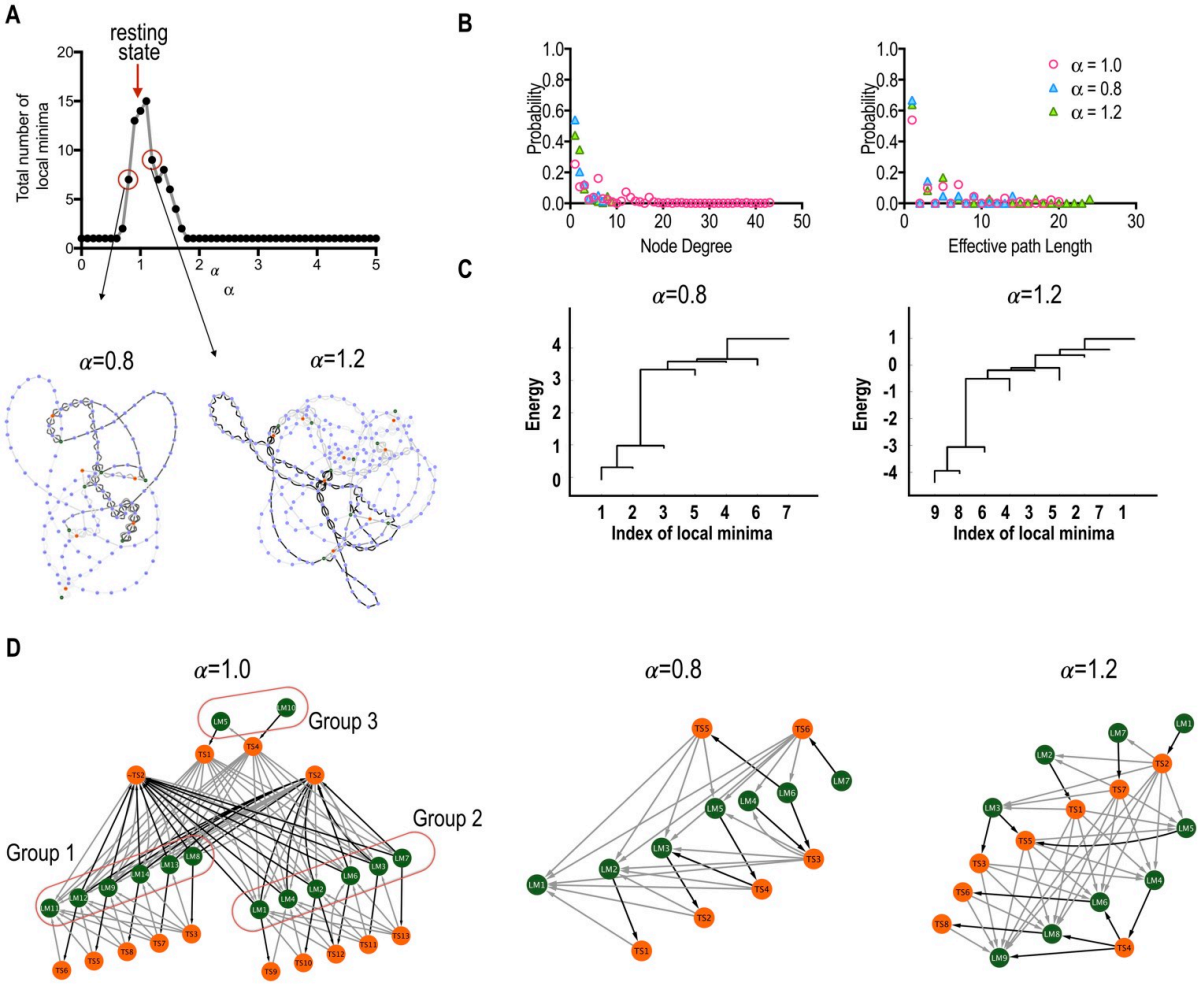
Comparisons between baseline and perturbed systems. (A) Total number of local minima in the perturbed systems, perturbed by changing global-scale pairwise interactions αJ_ij_. The state transition networks among full states (STN-FS) of two representative cases (α = 0.8 and 1.2) are shown in the lower panel. (B) Probabilities of the node degree and effective path length in the state transition network are plotted. The red, blue, and green colors represent α = 1.0, α = 0.8 and 1.2. (C) Local minima were clustered according to energy barriers. The leaf ends of the dendrogram represent the energy values of the corresponding local minima. (D) The state transition networks (STNs-LM) composed of rate-determining transition states (TS) and local minima states (LM) of two perturbed systems are presented. The green and orange colored nodes in the reduced state transition networks represent local minima and transition states, respectively.

The effects of the global strengths of pairwise interactions on the transition process were investigated by scaling all *J*_*ij*_ parameters (*αJ*_*ij*_, *α* = 0.0, 0.1, …, and 5.0). Both increases and decreases of the scale factor *α* tended to reduce the total number of local minima (Fig 5A). When a markedly small or large-scale factor, *α* < 0.7 or *α* > 1.7, was used, only one local minimum was found.

Here, we further analyzed two representative examples: *α* = 0.8 and *α* = 1.2. In both cases, the total number of local minima was reduced by perturbations, from 14 (baseline resting-state) to 7 (for *α* = 0.8) and 9 (for *α* = 1.2), and thus 21 and 36 transition processes were considered in their state transition networks, respectively.

In the state transition network of the weak coupling system (*α* = 0.8), 137 nodes and 258 edges, which were decreased compared to the baseline resting-state, were found. Under the strong pairwise interaction (*α* = 1.2), compared to the baseline resting-state, the total number of nodes was decreased (225 nodes) and that of edges was increased (456 edges). Since most pairwise parameters were positive, the energy of the states increased and decreased for weak and strong alterations in the scale parameter, respectively. In both cases, a positive correlation between node degree and energy was found for the nodes of the transient and transition states (Figure D in S1 File).

In the baseline resting-state, nodes were densely connected to other nodes (Figure D in S1 File), and, the maximum node degree was 44, which was larger than that of the altered systems (8 and 10, for *α* = 0.8, and 1.2, respectively). For all cases, more than half of the effective path lengths had a value of 1 (Fig 4). In the weak coupling system (*α* = 0.8), the longest effective path length was 15, which was smaller than that of the others (21 and 25 for *α* = 1.0, and 1.2, respectively).

In contrast to the baseline resting-state, in these altered systems, simple and deep energy valleys were found (Fig 5C). Indeed, the state transition processes were simpler than those of the baseline resting-state (Fig 5D). Except for LM6 in the weak coupling system, all local minima directly transitioned to their global minimum (Figure D in S1 File).

## 4. Discussion

The brain at rest has been considered a highly dynamic complex system operating at a critical value of coupling that maximizes multistability [21, 34–36, 55]. Beyond the multistability of the resting-state cortical system, we systematically investigated the architecture of the state transition processes by applying a graph-theoretical analysis to state transition. State transition network analysis suggests a well-organized state transition process embedded in the resting-state human cerebral cortex system. The characteristics of the state transition in the resting-state cortex system are discussed in the subsequent paragraphs.

### 4.1 Intermediate states in brain state dynamics

**Fig 6:**
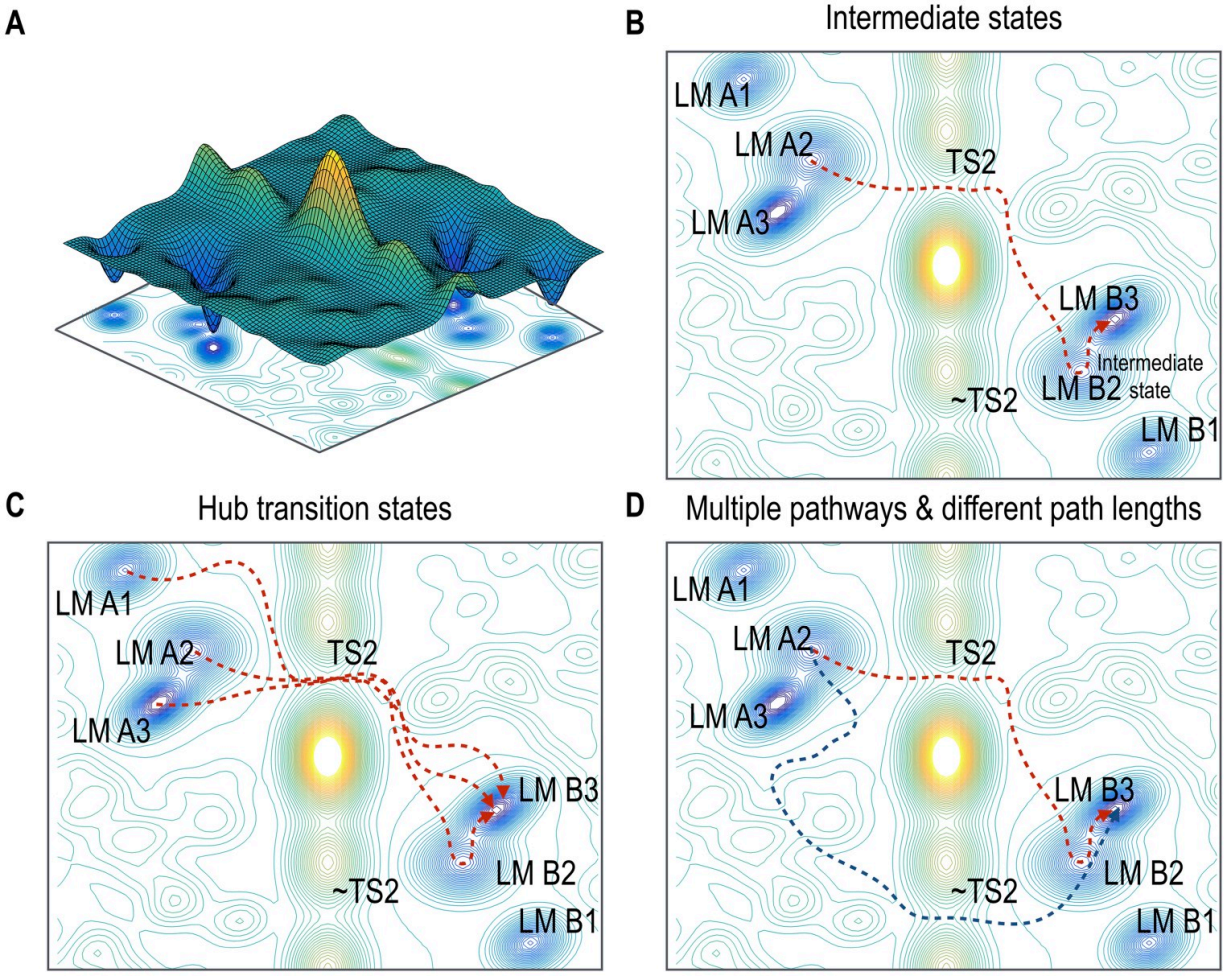
Illustrative analogy of the state transitions in the cerebral cortex system at rest. (A) A schematic energy landscape contains six stable states, which are classified into two major groups. (B) A representative pathway of the state transition is shown. Several stable states (local minima) operated as intermediate states of the state transition processes. (C) The hub transition state (saddle point) TS2 mediates the inter-group transition process between stable states in the two groups. (D) ~TS2 also operated as an alternative hub transition state (saddle points) when TS2 was removed. This suggests multiple pathways and indicates redundant mechanisms of state transitions. Since the energy difference between TS2 and ~TS2 is small, transition rates of the two pathways, colored red and blue, are similar.

The resting-state brain has intermediate states in state dynamics. Some state transitions in the cerebral cortex system toward the global local minimum occurred in multi-steps via several intermediate stable states (or intermediate local minima) (Figs 2C and 6B). This phenomenon is similarly found in biochemical reactions by enzymes in biological systems [30, 56–59]. For instance, during the rebinding of ligand (CO or O_2_ molecules) to myoglobin after photolysis, several intermediate states were observed in spectroscopic experiments, and these intermediates have often been explained in terms of the regulation of ligand binding mechanisms [57–59]. The state transitions during membrane fusion processes occur via intermediate states, which were explored in computational and experimental studies [60–62]. Similar to these phenomena in biochemical systems, the intermediate stable states during brain state transition may also play a role in lowering energy barriers. We speculate that this lowered energy barrier may regulate and expedite transitions along certain pathways of brain state transitions in the resting-state whole-brain system. It should also be noted that transitions between some pairs of local minima are straightforward without any intermediate transition states.

### 4.2 Characteristics of brain state transitions in the resting-state dynamics

Current network analysis of stable states of the cerebral cortex suggests several characteristics of brain state dynamics.

First, in the brain state transition network, local minima are highly clustered mainly into two groups, and the manner in which state transitions among distributed local minimum occurred was different between inter-group and intra-group transitions.

Second, most inter-group state transitions (from a local minimum at a cluster to a local minimum at the other) occurred via some hub transition states (saddle points) (e.g., TS2 and ~TS2) in the transition pathway (Fig 6D). This phenomenon makes the inter-group transition different from the intra-group state transition, where the transition state along the path between two states differed according to the initial state. Those hub transition states are analogous to hubs found in the conventional network analysis of the brain connectome. Brain connectome studies have shown a hub-like structural architecture in the brain, which is considered to mediate efficient information exchange [39–42]. Similar to network analysis focusing on the spatial geometry of the connectome, the current result suggests that inter-group brain state transitions occur mostly via hub states (more frequently occurring states) in the temporal geometry (Fig 6C).

Third, path lengths (number of transitions to reach the target state) were positively correlated with the Hamming distances (i.e., number of different state bits (or regions)) between the initial and final states within the intra-group transitions. This result implies that the transition states of intra-group transitions take advantage of shorter transition paths, i.e., efficient transition from a brain state to the other state with minimal transition numbers. In the inter-group state transitions, path lengths were determined by the Hamming distance between the initial state and the hub transition state on the path, not the final state (Figs 4D, 4E, 4F and 6D). We speculated that for significant state changes in the cortical system, the brain may minimize transitioning costs by traveling via hub transition states, not simply following short transition paths.

### 4.3 Redundant inter-group state transition in the cortical system

The current simulation study suggests that the cerebral cortex has redundant transition pathways. A transition state (e.g., TS2) mediates most of the inter-group state transition processes, serving as a transition hub in the resting-state transition network (STN-LM). When we excluded this hub transition state, its complementary state ~TS2 appeared to serve as a detour for inter-group transitions with similar transition rates. This alternative hub ~TS2 was the complementary state of the hub state (TS2) of the baseline resting-state. The energy level of ~TS2 was similar to that of TS2 and the rates of the transition processes were similar to each other. We considered pathways via ~TS2 as “redundant” pathway in inter-group transition processes, as those were chosen after removing TS2 as a transition state.

The existence of multiple pathways has been reported in some complex systems where a reaction occurs among multiple units cooperatively. For example, in some biomolecule systems, e.g. F1-ATPase and myosin V motor, two reaction pathways have been reported [63, 64]. Multiple transition pathways may be associated with “degeneracy” or “redundancy” in the complex brain system [65, 66]. The redundant pathways could be particularly advantageous in maintaining effective state transitions when a certain transient state cannot play its role in the state transition.

### 4.4 Baseline brain state dynamics compared to those of altered systems

To understand the organization principle of the baseline configuration for brain state dynamics as done in Kang et al. [21], we compared the baseline (observed) network configuration with virtual (artificially altered) network configurations by scaling the pairwise interactions, and analyzed their state transition dynamics. Compared to the virtual networks, the baseline resting-state transition network (STN-FS) contained a bigger number of states with high node degrees and relatively longer path lengths. Furthermore, neither the clustered structure nor the intermediate states of the baseline system were observed in the altered virtual systems. For example, in the STN-LM of the baseline system, which contains nodes on the pathway toward the global minimum (LM11) from other local minima (Fig 2), four types of transition processes were identified with four local minima (LM7, LM9, LM12, and LM14) as intermediate states. This property was not found in the virtual systems, which showed a much simpler transition network (Fig 2 and S3). We also conducted energy landscape analysis of network configurations with random values and with randomly permutated *H*_*i*_ and *J*_ij_. The results presented in the Supporting Information suggest simpler energy landscape and simpler transition network in the random network configurations than in the real resting-state network (see Section C in S1 File).

Furthermore, hub intermediate states and hub transition states were found only in the baseline resting-state system, not in the virtual systems. In the virtual systems, altered by scaling pairwise interactions from the baseline resting-state system, local minima having a high node degree disappeared; the hub-like local minima of the baseline system (having relatively high energy) were eliminated first by scaling pairwise interactions. This phenomenon, of which higher energy was eliminated first by network alteration, was consistent with that reported in our previous study on the subcortical system [21]. Meanwhile, low-energy local minima tended to persist even after network alteration. If we consider some task performances as states deviating from the baseline system, those sustaining local minima may act as common bases from which diverse functions arise or as fundamental elements of maintenance of dynamic brain systems. It may be that the baseline cerebral cortex system is configured to allow network systems to effectively transition among diverse brain states, which may be a necessary element in the workings of the complex network systems exhibiting multiple stable states.

All these transition characteristics may possibly be embedded in the nonlinear coupling over the structural network. We speculate that network topology may provide a biased playground of multistability, and endogenous fluctuations during resting-state may drive state transitions over the structural playground. This interpretation about the interplay between network topology and noise is in line with the dynamic nature of the brain [9, 21]. In the current study, we showed that the multistable nature of brain states and the well-organized properties of the transition processes can emerge from nonlinear interactions over the cortical brain network.

### 4.5 Nonlinear dynamic brain systems

Resting-state brain dynamics and non-stationary functional connectivity have recently been explored by evaluating brain connectivity in sliding window fashion from the viewpoint of a linear system [11–20]. For example, Park and colleagues assumed linear interactions (connectivity) between brain regions change by time, and these time-dependent interactions were estimated for consecutive windows [12]. In contrast, multistability in the complex system is an emergent property of nonlinear interactions among nodes in the system, without any changes in the internal connectivity. From the perspective of the nonlinear system, Hansen, Battaglia [37] suggested that non-stationary functional connectivity (FC) (particularly, rapid transitions switching between a few discrete FC states) can be explained by the non-linearity of the nodal activity that derives the structural brain system. Spiegler, Hansen [67] attributed the nonstationary FC to the criticality of the nonlinear brain system embedded in the structural network topology. Similarly, Cabral et al. also showed that dynamic functional connectivity can emerge from a static structural connectivity with various non-linear dynamic models of the brain [55]. They showed that diverse FC states are emergent when the brain is operating at the edge of criticality. Pillai and Jirsa [68] also showed that multiple sub-states undergo structured flows on the manifold of the low-dimensional state spaces (functional subspaces) and this emergent behavior is attributable to the synaptic coupling level over the nonlinear interactions. Rabinovich and colleagues [69] argues that both flexible and reproducible transitions among multiple meta-stable states can emerge in the nonlinear system, which may explain state transitions in the decision-making process. All those studies [37, 55, 67–69] are based on a model with nonlinear temporal dynamics described using a differential equation. In terms of the nonlinear interaction and its consequent emergence of multiple states in the complex brain, our approach using pairwise MEM is in line with those studies [37, 55, 67–69]. However, the current evaluation differs from those studies in that the analysis of brain dynamics with a pairwise MEM is based on the statistical mechanics, which deals with the emergence of stable states and their transitions in terms of probability. Nevertheless, the two approaches (analysis with a differential equation and analysis in the statistical mechanics) are known to be equivalent since ensemble probability distributions of each state (in statistical mechanics) can be derived from a large number of trajectories as solutions of a differential equation (for a rigorous explanation, see ergodic theorem in the statistical physics).

A small number of major transition paths in the current study may serve as manifold-like transitions found in Pillai and Jirsa [68], where a lower dimensional manifold of state transitions was induced by an asymmetric interaction (due to task). The organized state transitions explored in this study may be correspondent to reproducible transitions among multiple meta-states in Rabinovich et al. [69].

### 4.6. Neurobiological interpretation

**Fig 7:**
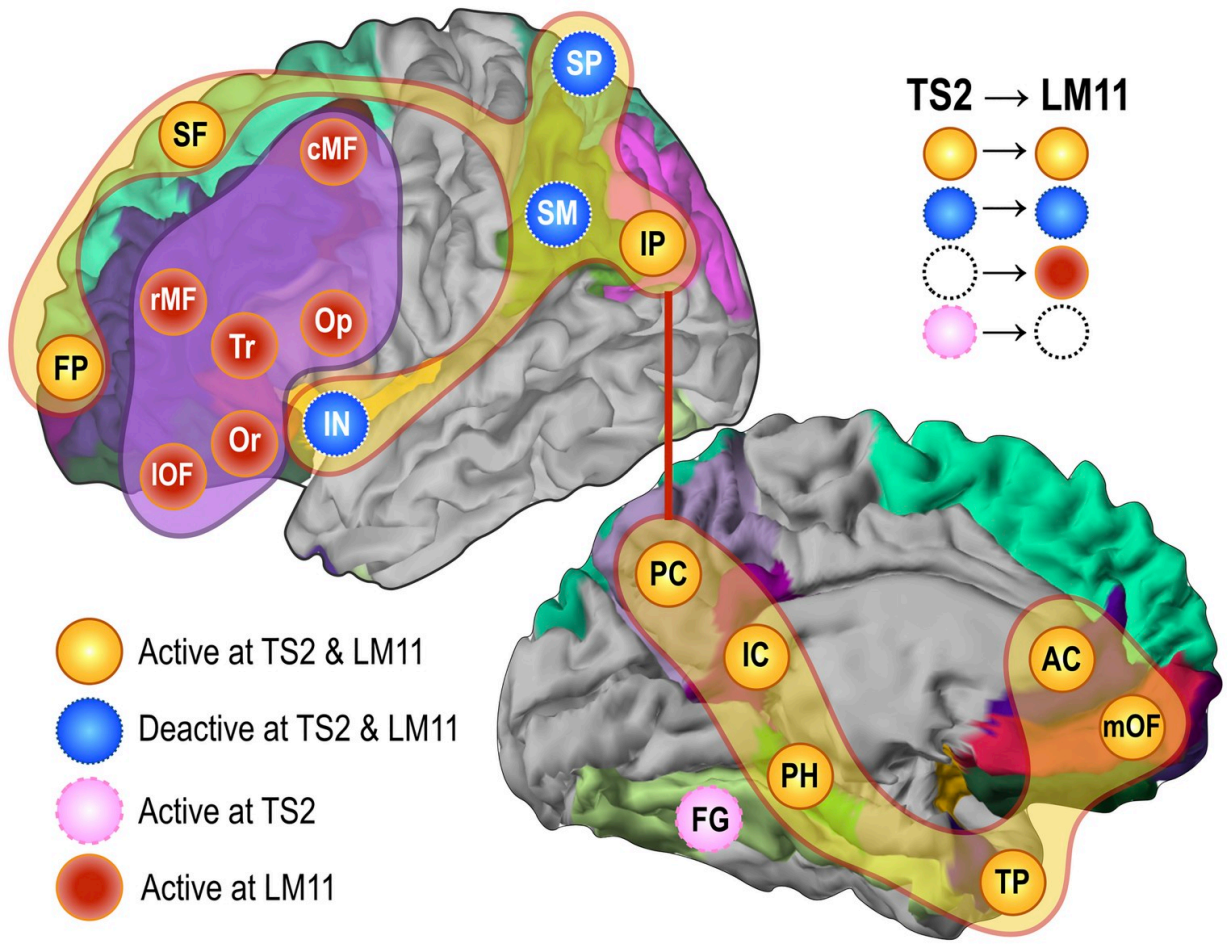
The hub transition state TS2 and the global minimum LM11. The hub transition state (saddle point) TS2, corresponding to the typical default mode network in (70), has a distributed pattern of activations at the posterior cingulate cortex, medial prefrontal cortex, anterior cingulate cortex, superior frontal cortex, frontal pole and the inferior parietal lobe and deactivations at the superior parietal cortex, supramarginal gyrus and insula. The global minimum LM11 comprises combined activations of TS2 and the middle and inferior prefrontal cortices. The fusiform gyrus takes part in TS2 but disappears in LM11. Meanwhile, the prefrontal brain regions appear to be active in the global minimum LM11.

In this study, the transition state TS2 appears to mediate most transitions, particularly from inter-group transitions between complementary states. Neurobiologically, TS2 (Fig 7) corresponds to the typical default mode network as shown in [70, 71]. In particular, the DMN in [70] has a distributed pattern of activations at the posterior cingulate cortex, medial prefrontal cortex, anterior cingulate cortex, superior frontal cortex, frontal pole and inferior parietal lobe. Many of those DMN regions are known to play hubs in the structural brain network [72]. The current transition network analysis newly suggests that DMN also serves as a transition hub in the state transition. In the nonlinear systems perspective, the brain has been considered to be in a saddle point of phase transition, ready to perform diverse tasks rather than in a stable state [21, 34–36, 55]. In this respect, it is not surprising that DMN, a core of the brain network, mediates most state transitions.

The global minimum LM11 represents a combined activation of TS2 (DMN) and the middle and inferior prefrontal cortices. Why the coactivation at the prefrontal and default mode networks appears frequently is an open question but some previous works support this phenomenon. The distributed metabolic activity pattern in a group average fluorodeoxyglucose (FDG) positron emission tomography (PET) in resting state [73] is highly consistent with the pattern of LM11. Considering that FDG-PET indicates accumulated energy metabolism of the brain at rest, increased metabolism at brain regions corresponding to LM11 explains frequent occurrence of LM11, i.e., global minimum. The coactivation among distributed regions of the DMN and prefrontal cortices may be attributable to anatomical neural projections. For example, the regions of DMN are highly interconnected via anatomical projections observed in fiber tracking of diffusion tensor imaging [74].

Note that TS2 (i.e., DMN) mediates sequential state transitions particularly between complementary states (in polarity). When brain states in Group 2 (Fig 3) transit to their complementary states in Group 1 or eventually to the global minimum state (LM11), those states progressively (but statistically) transit to destinations via the DMN transition state (TS2). This polarity switching has also been observed in previous MEM studies [25, 26, 75]. All this transition occurs progressively, via transition states or intermediate local minima. The progressive spatio-temporal transition may be associated with appearance of microstate modes detected in EEG [76] and some phenomena of travelling waves [77, 78] or metastable waves [79]. Considering (semi-)cyclic waves of spatial activation patterns during resting state [76, 78, 79], the progressive transition between complementary states or global minimum is not surprising.

The local minima found in the current study may be associated with diverse introspective thoughts or unconscious reactions to intrinsic sensations and the state transitions among local minima may reflect mind-wandering. Although the functional role of each local minimum and the mechanism for state transition are unknown, the anatomical configuration of the brain network may contribute to form the typical energy landscape of brain states, which induces organized transitions in this study. All these speculations need to be further researched.

### 4.7 Limitations and challenges

The current study has several limitations and challenges. Due to the high computational cost and the requirement of a large sample size, we evaluated the dynamics of a reduced brain system at each hemisphere (focusing on the left hemisphere in the main text) but did not evaluate those of the whole-brain system. In spite of strong symmetry (e.g., 21), the interaction between two hemispheres and its effect on the dynamic system in the whole-brain system remain to be explored. For the same reason, we did not include sensory and subcortical brain regions in the current analysis. We speculate that the dynamic properties of the whole-brain nonlinear system would be much more complex. In spite of technical challenges, exploration of the state transition properties of the whole-brain system with more precisely parcellated brain regions would greatly expand our understanding of the brain system.

In the preprocessing step, we decided to conduct a global regression since we focus on the dynamics of regional brain states induced by pairwise interactions within the system (not induced by any other modulatory effects) in an equilibrium. To define a regional brain state with an activity of a region that fluctuates locally, we applied MEM to fMRI signals after global regression. The global regression emphasized properties of the state dynamics when it was applied to the subcortical brain system in our previous study [21].

Like any other modeling approach, the modelling with MEM simplifies the dynamic phenomenon of the brain by binarizing regional activities into two binarized states, “1” and “0”. This simplification may not be precise enough to describe microlevel states at a region but this simplification enables us to explore nonlinear spatio-temporal dynamics of the complex brain system, which may otherwise not be easily achieved. Since binarization of regional signals was done after global regression, the regional states “1” and “0” are defined relative to the global mean at each time point. Thus, we denoted “0” as deactive (suppressed) compared to the global mean although it may denote inactivation at some regions. Nevertheless, the neurophysiological underpinnings are not clear whether the low fMRI amplitudes (denoted as “0”) indicate inactive (silent) or deactive (suppressed) neural states. Due to the complex neuro-vascular coupling in the BOLD-fMRI [80], even high fMRI amplitudes (denoted as “1”) can possibly be induced by increased metabolic demands after firing of inhibitory populations (a suppressed brain state). Therefore, the definition of brain states should be further clarified with more pieces of knowledge on the neural states reflected in the fMRI signals.

Despite this simplification (binarization), the pairwise MEM provides a new framework of brain research by considering the distributed brain activity as a result of nonlinear pairwise interactions among brain regions in a system. Some computational models have nonlinear formulations of interactions comparable to the pairwise MEM. Deco and colleagues [81] introduced a nonlinear dynamic brain model based on a dynamical mean field reduction. The method in [81], however, does not estimate interaction parameters directly. Instead, they simplified the model by adopting structural connectivity as interaction parameters [81]. In the task-based fMRI analysis, a nonlinear dynamic causal model has been proposed [82]. The nonlinear dynamic causal model [82], which is practically limited to a small size circuit with an external perturbation (input), does not provide a direct way of evaluating the probability of state occurrence during resting state.

In contrast to these nonlinear approaches to the brain, Vidaurre and colleagues [83, 84] have introduced a hidden Markov modelling (HMM) to identify brain states and to explore state transitions in the brain. However, a state in the HMM is defined in terms of a whole-brain connectivity pattern (embedded in the cross-spectral density, corresponding to the system parameter J_ij_ in the MEM) that changes along the time course. Similarly, the dynamic nature of the brain has been explored in terms of temporal changes in its functional connectivity [11–20]. Meanwhile, MEM assumes a nonlinear system that generates multistablity without changes in the system configuration and the state transition is a result of nonlinear interactions. In this respect, MEM is a practical way of exploring the brain’s complex dynamics, which is not easily replaced. In line with this notion, many studies have shown the usefulness of MEM in the analysis of brain dynamics with respect to a nonlinear system [21, 25, 26, 75, 85].

Previous studies have revealed alterations in the dynamics of networks associated with brain disorders such as schizophrenia [86, 87] and autism [75]. A growing number of studies are showing altered dynamics in other brain disorders as well. However, the dynamic properties in brain diseases have not been thoroughly researched. The current frameworks for dynamic brain states can be used to identify altered dynamic architectures in neuropsychiatric disorders. Research using clinical data will yield results that can validate the usefulness of the proposed approach.

## Supporting information

S1 File Supporting information file. Supplementary figures, results of state transition network analysis of the resting-state cerebral cortex system in the right-hemisphere, and Energy landscape of randomized system are included.
(PDF)
