# Supplementary material for: Graph-theoretical analysis for energy landscape reveals the organization of state transitions in the resting-state human cerebral cortex
Source: PLoS One. 2019 Sep 9;14(9):e0222161. doi: 10.1371/journal.pone.0222161 (PMC6733463; doi:10.1371/journal.pone.0222161)
Supplement: S1 File — Supplementary figures, results of state transition network analysis of the resting-state cerebral cortex system in the right-hemisphere, and Energy landscape of randomized system are included. (PDF) [file pone.0222161.s001.pdf]

Supporting Information

Graph-theoretical analysis for energy landscape reveals the organization of state transitions in the resting-state human cerebral cortex

Jiyoung Kang[1,2], Chongwon Pae[2], and Hae-Jeong Park[1,2,3,4] *

[1]Center for Systems and Translational Brain Sciences, Institute of Human Complexity and Systems Science, Yonsei University, Seoul, Republic of Korea
[2]Department of Nuclear Medicine, Yonsei University College of Medicine, Seoul, Republic of Korea
[3]BK21 PLUS Project for Medical Science, Yonsei University College of Medicine, Seoul, Republic of Korea
[4]Department of Cognitive Science, Yonsei University, Seoul, Republic of Korea

Contents

A. Supplementary figures
B. State transition network analysis of the resting-state cerebral cortex system in the right-hemisphere
C. Energy landscape of randomized system

A. Supplementary figures

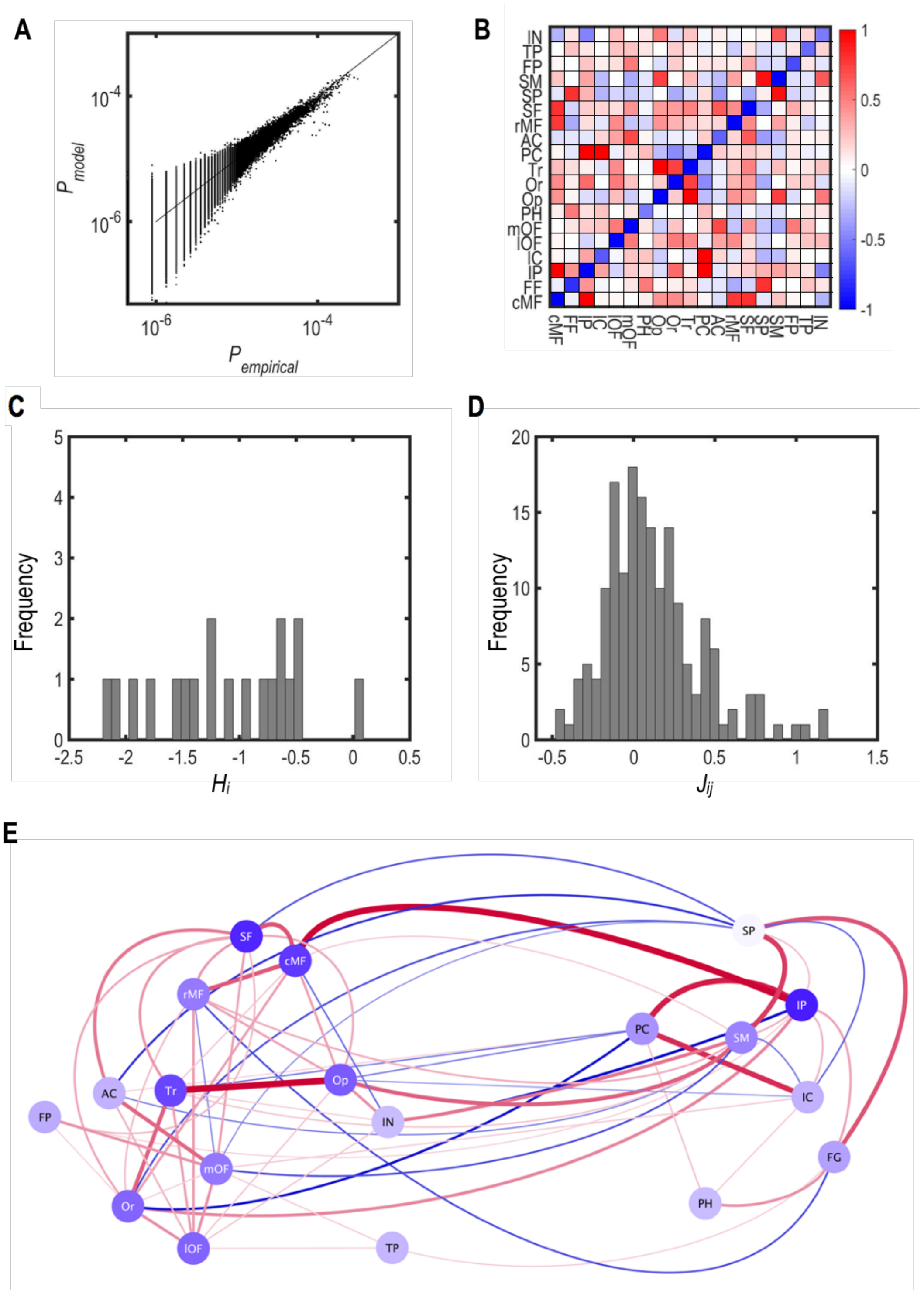

Figure A. Results of the pairwise MEM for resting state of the cerebral cortex system. (A) The probabilities of the states observed using the constructed MEM were compared with empirical data. (B) Estimated MEM parameters, baseline sensitivity (H_i), and pairwise interaction (J_{ij}) parameters are shown. Diagonal elements represent H_i. Histograms of MEM parameters are shown for H_i and J_{ij} in (C) and (D), respectively. (E) Resting-state cerebral cortex networks

estimated by the MEM are summarized. Among all pairwise parameters of MEM J_{ij}, only $|J_{ij}| > 0.2$ were displayed. The thickness of the lines represents the strength of the given interactions. The red and blue lines represent negative and positive J_{ij} parameters, respectively. Most of the ROIs were inactivated by negative H_i values (Figure S1B and S1C). The pairwise interactions, J_{ij}, were distributed among positive and negative values, ranging from -0.4807 to 1.1950 (Figure S1B and S1D). The baseline sensitivities of the inferior parietal lobe, superior frontal gyrus, caudal middle frontal gyrus, and pars-triangularis (H_{IP}, H_{SF}, H_{cMF}, and H_{Tr}) were more negative than those of other regions. Four pairwise interaction parameters, $J_{\mathrm{Op\text{-}Tr}}$, $J_{\mathrm{cMF\text{-}IP}}$, $J_{\mathrm{IP\text{-}PC}}$, and $J_{\mathrm{IC\text{-}PC}}$, were relatively larger than others. Thus, each ROI of the system would be inactivated without pairwise interactions. Estimated strong positive and negative pairwise interactions reflect how two nodes were easily co-activated, and the activation patterns of the local minima (Fig. S1E). The superior parietal (SP), isthmus cingulate (IC) and precuneus (PC) were positively and negatively connected with other ROIs.

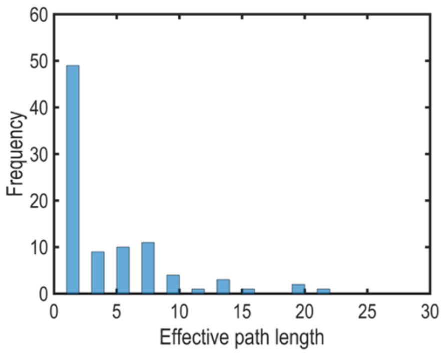

Figure B. Results of the entire state transition network. Histogram of the effective path length is shown.

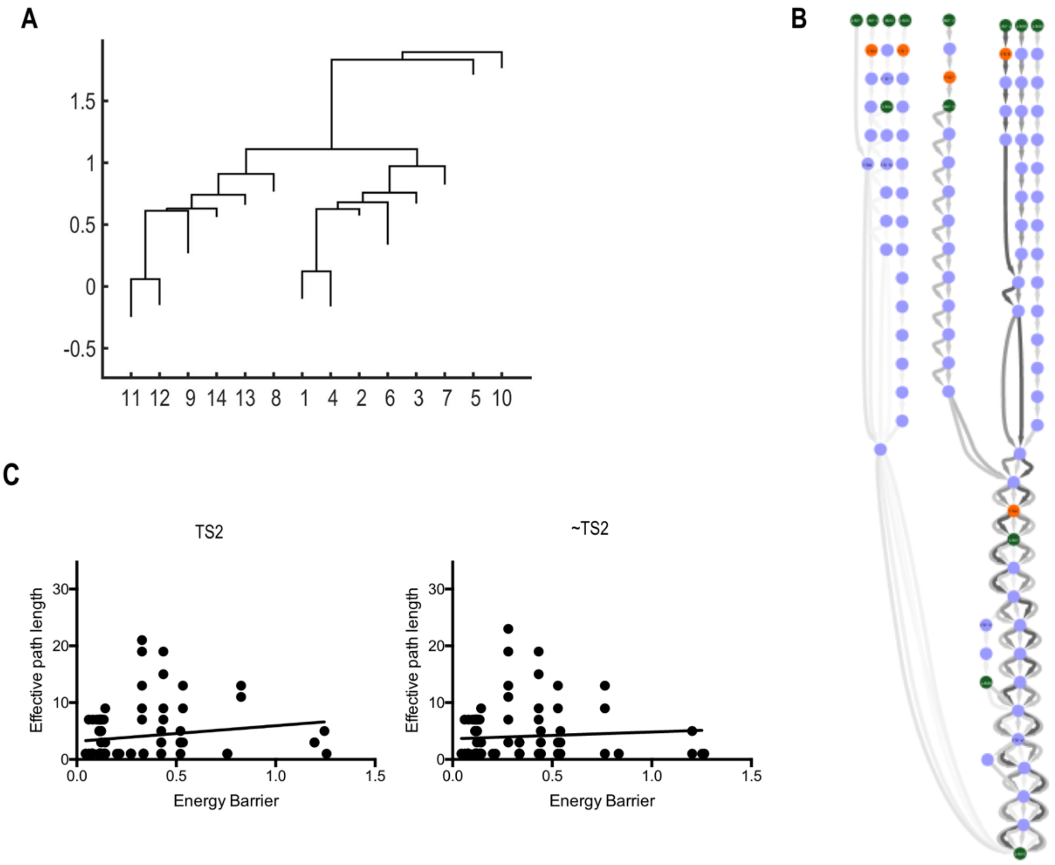

Figure C. Analysis of state transition network of ~TS2 system. (A) Clustered local minima using energy barriers as the distance metric. (B) The state transition network that relate to transition process to the lowest local minimum is presented. All states which appeared in the state transition process were assigned nodes. Orange, light blue, and green color represent transition, transient, and local minima states, respectively. (C) Both of TS2 and ~TS2 systems did not show any correlation between the energy barrier and effective path lengths.

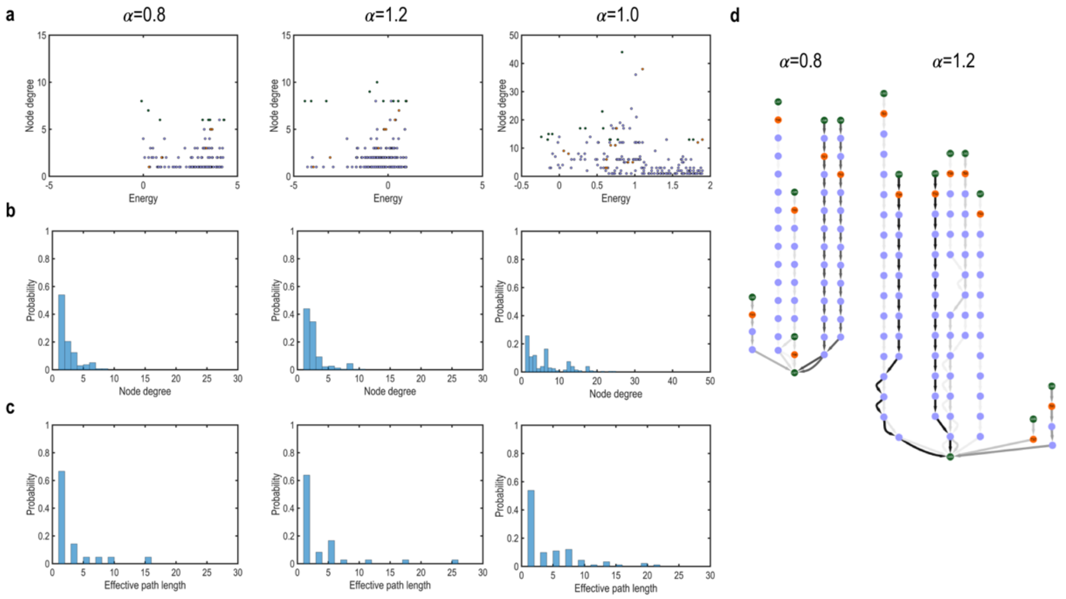

Figure D. Network properties of the entire state transition networks (ESTNs) for baseline and perturbed systems. (A) Node degree and energies of the nodes in the state transition networks are plotted. (B, C) Histograms for the node degree and effective path length are shown in (B) and (C), respectively. The left and middle panel show results of the perturbed systems, α = 0.8 and 1.2, respectively. The right panel shows results of baseline resting state (α = 1.0). (D) The global minimum focused transition networks (GFTNs) for the perturbed systems (α = 0.8 and 1.2) are shown.

B. State transition network analysis of the resting state in right-hemisphere

In the main text, cortical regions in the left-hemisphere were mainly investigated. To confirm if brain dynamics in the right-hemisphere contain similar properties, we further constructed the maximum entropy model (MEM) for the right-hemisphere. The activation patterns of rs-fMRI data were reproduced with a high accuracy of fit (r_D = 85.5 %) and reliability (ER = 99.9 %) (Figure S5A). Baseline sensitivity parameters H_i and pairwise interaction, J_{ij}, are displayed in Figure S5B. Strong positive correlation was observed between the estimated MEM parameters of the right-hemisphere and the left-hemisphere (r=0.982, p=7.932 × 10^{-137}). Although most of MEM parameters of the right-hemisphere were similar to those of the left-hemisphere, several MEM parameters were different; e.g., H_{SF}, H_{rMF}, and $J_{IP\text{-}rMF}$ (Figure S5C).

Analysis of energy landscape identified 18 local minima (having lower energy than their neighbor states) of the right-hemisphere cerebral cortex system at rest. From analysis of the state transition network among full states (STN-FS) and state transition processes (STN-GM) from local minima (LM) toward the global local minimum (LM15), we confirmed that similar properties of state transitions such as existence of hub nodes and multistep process were conserved in the resting-state cerebral cortex system of the right-hemisphere (Figure S6). Activation patterns of local minima and transition rates were similar between the right and left cerebral cortex systems, and clustered (well organized) state transition processes was also identified in the right-hemisphere cerebral cortex system (Figure S7). More specifically, we identified three groups, and similar to the left-hemisphere cerebral cortex system at rest, we found TS1 which appears to mediate transition between two large groups. When we exclude this hub state, its complementary state ~TS1 appeared to serve as a detour for inter-group transitions with similar transition rates (99 %). Thus, "redundant" pathways in inter-group transition processes existed in both the left and right cerebral cortex systems.

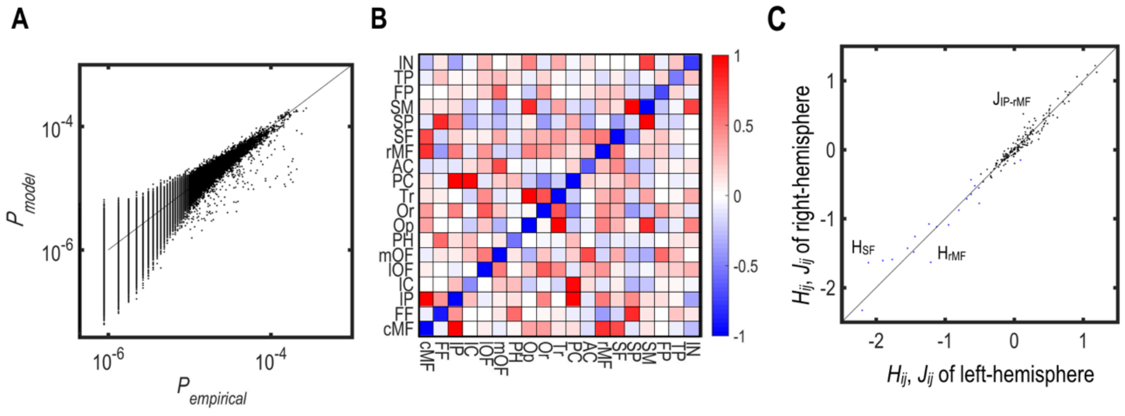

Figure E. Results of the pairwise MEM for the resting state of the cerebral cortex system of right-hemisphere. (A) The probabilities of the states observed using the constructed MEM were compared with empirical data. (B) Estimated MEM parameters, baseline sensitivity (H_i), and pairwise interaction (J_{ij}) parameters are shown. Diagonal elements represent H_i. (C) Estimated MEM parameters were similar to those of left-hemisphere. (r=0.982, p=7.932 × 10^{-137}). Here, H_i and J_{ij} were colored by blue and black, respectively.

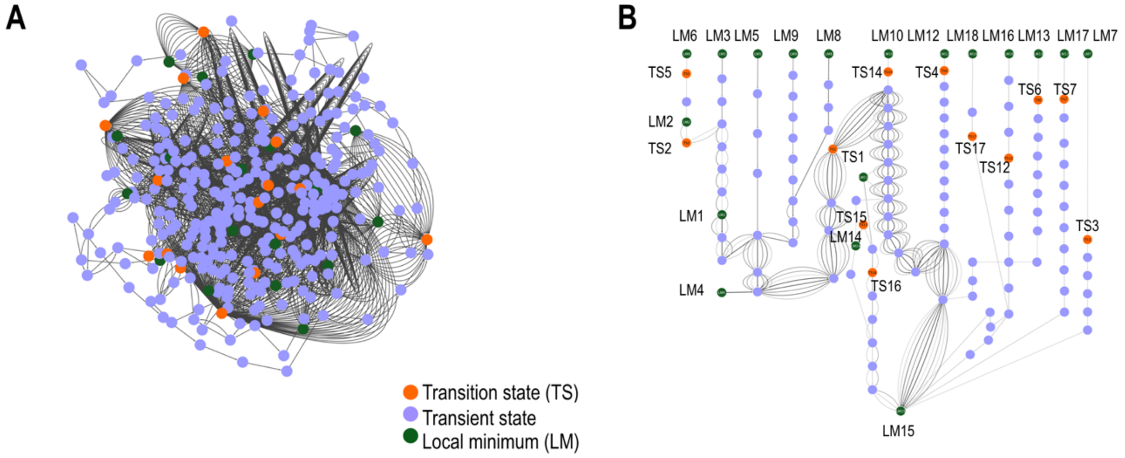

Figure F. Analysis of the state transition networks (right-hemisphere). (A) The state transition network among full states (STN-FS) of the right-hemisphere is shown. We assigned all states in the state transition process to the nodes. (B) State transition processes (STN-GM) from local minima (LM) toward the global local minimum (LM15) is shown in (B). The green, blue, and orange colors represent local minima, transient, and transition states, respectively.

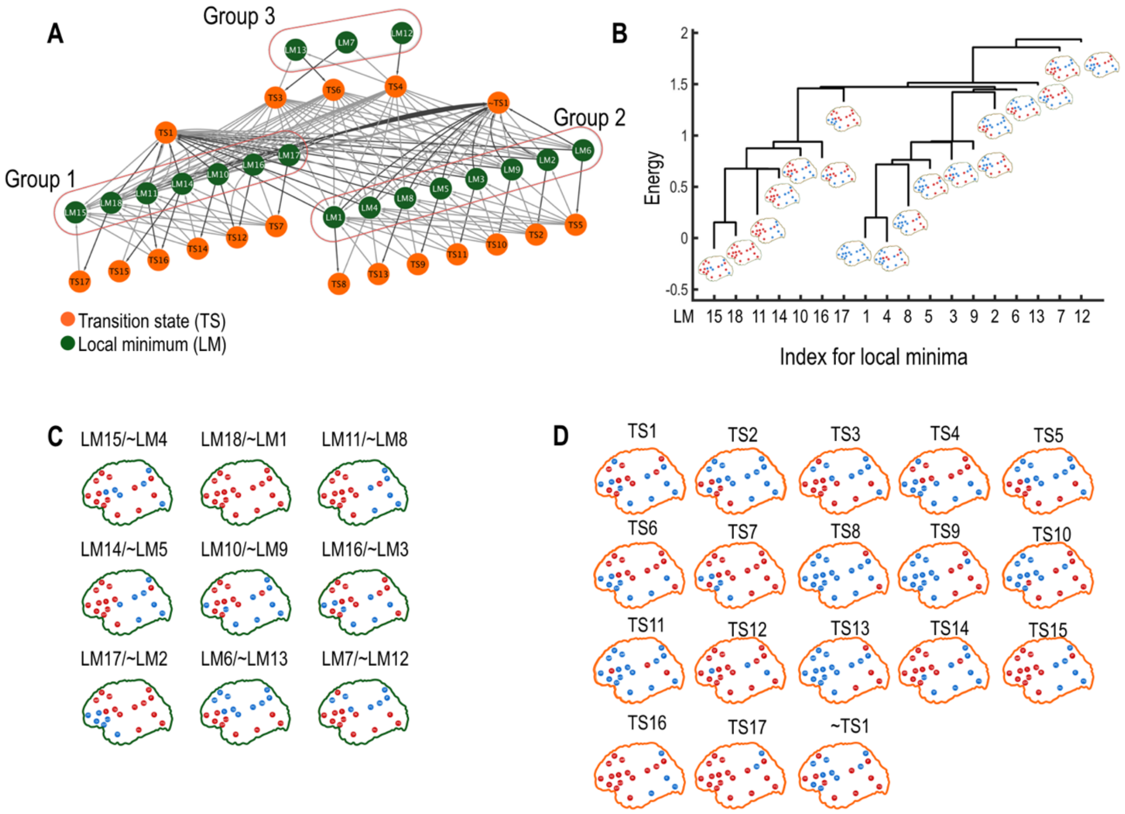

Figure G. Analysis of the state transition network (STN-LM) composed of rate-determining transition states (TS) and local minima states (LM) (right-hemisphere). (A) The STN-LM is shown. Black and gray colored lines represent in- and out- processes from the state transition states. (B) Local minima (LM) were clustered according to energy barriers. The leaf ends of the dendrogram represent the energy values of the corresponding local minima. (C) Activation patterns of the local minima. The "~" sign represents complementary states. For instance, LM15/~LM4 indicates that LM2 and LM14 are each other's complementary states. (D) Activity patterns of the transition states are shown with TS1 and ~TS1 as major hub transition states. The red and blue dots represent the active and inactive states of the ROIs. The green and orange colors represent local minima and transition states.

C. Energy landscape of randomized system

To show the characteristic of the resting state brain network, we generated and evaluated 500 randomized MEMs (sets of MEM parameters) in two ways.

First, we generated 500 MEM parameter sets by choosing random values within a range of [-2.2, 0.1] for H_i and a range of [-0.5, 1.2] for J_{ij}. These ranges were determined by the minimum and maximum values of the currently estimated MEM parameters from the resting state fMRI. By using energy landscape analysis, we found that the total numbers of local minima from randomly generated MEMs were less than three (supporting information Figure S8A), which are extremely smaller than that of the resting state brain (n = 14).

Second, we generated another 500 MEMs, by permutating H_i and permutating J_{ij} parameters (similar to a study in Watanabe et al (2013)). As a result, we found that most systems have smaller numbers of local minima (the mean was around 5) than that of resting state fMRI. We did not find an organized structure in the state transition network of energy landscapes from those permuted MEMs, as shown in the perturbation analysis exampled in Figure 5 (C) and (D) with $\alpha = 0.8$ and $\alpha = 1.2$. Even in some systems with total numbers of local minima similar to that of experimental data, their energy landscapes were simpler than that of resting state fMRI (see below supporting information Figures S8B and S9). We presented a simulated system which contains five local minima as a representative example of random MEMs. The system has a simple transition network structure (no clustered or organized structure similar to that of Figure 5) (supporting information Figure S9).

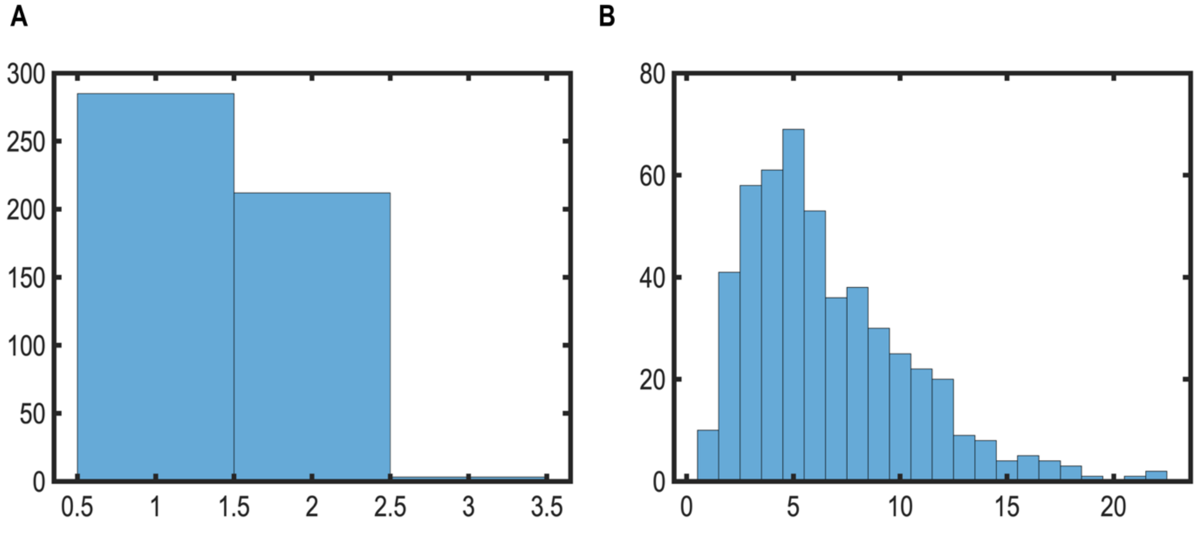

Figure H. Histogram of the total number of local minima of randomized (A) and shuffled systems (B)

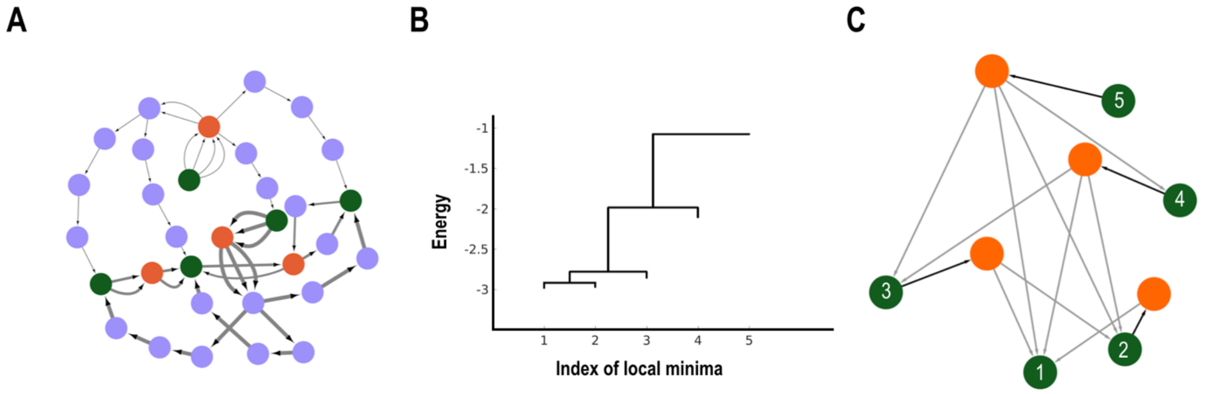

Figure I. A representative example of state transition network. Entire state transition network (A), dendrogram of energy landscape (B), and state transition network among rate-determining transition states and local minima states (STN-LM)
